# Impact of music on anxiety and pain control during extracorporeal shockwave lithotripsy: A protocol for systematic review and meta-analysis

**DOI:** 10.1097/MD.0000000000023684

**Published:** 2021-01-29

**Authors:** Zhenghao Wang, Dechao Feng, Wuran Wei

**Affiliations:** Department of Urology, Institute of Urology, West China Hospital, Sichuan University, Chengdu, China.

**Keywords:** anxiety, extracorporeal shock wave lithotripsy, meta-analysis, music, pain, urolithiasis

## Abstract

**Background:**

The present evidence is insufficient for evaluating the impact of exclusive music therapy on anxiety and pain control in extracorporeal shock wave lithotripsy (ESWL).

**Methods:**

A systematic review and meta-analysis was conducted to explore the efficacy of music therapy in reducing pain and anxiety in patients undergoing ESWL. PubMed, Web of Science, Embase, EBSCO, and Cochrane library databases (updated March 2020) were searched for randomized controlled trials assessing music therapy in reducing pain and anxiety in patients undergoing ESWL. The search strategy and study selection process were managed according to the Preferred Reporting Items for Systematic Reviews and Meta-analysis statement.

**Results:**

Five randomized controlled trials were included in the meta-analysis. Overall, music intervention groups experienced significant reductions in pain (risk ratios = –1.20, 95% confidence intervals = –1.95 to –0.45, *P* = .002) and anxiety (risk ratios = –3.31, 95% confidence intervals = –4.97 to –1.84, *P* < .0001) compared with control groups during ESWL. Music therapy gave patient more satisfaction with the treatment and a willingness to repeat the therapy was reported. However, there was no significant difference in the stone clearance rate.

**Conclusions:**

Listening to music can reduce patient's pain and anxiety significantly with increased therapy satisfaction and willingness to repeat.

## 1 Introduction

Calculi in the urinary tract is a common disorder and affects nearly 12% of the population worldwide.^[1]^ Urinary stones often cause haematuria, renal colic, vomiting, and nausea for acute blockages. Chronic obstruction of the urinary tract can lead to hydronephrosis and even renal failure.^[2]^ Among all current surgical treatments, extracorporeal shock wave lithotripsy (ESWL) is the most frequently used in clinical practice since the 1980s due to its high efficacy and low incidence of complications.^[3]^

However, a major issue affecting patients after ESWL treatment most is pain and anxiety. According to previous studies, the pain may be derived from 2 sources. Firstly, the increased pressure within the kidney and secondly, the trauma in the skin and muscles and the stretching of the renal capsule.^[4,5]^ Furthermore, the patients’ tolerance and the effectiveness of this procedure is highly affected by both ESWL-related pain and anxiety.^[4,6]^ To reduce the levels of pain and anxiety and increase the compliance of the patients, various complementary therapies such as anesthesia (including general and spinal anesthesia) or other pharmacological options (including sedative agents, opioids, or analgesics) were introduced.^[7]^ Although these approaches have been proven effective, it is still not highly recommended due to the associated side-effects and costs.^[8]^

Therefore, nonpharmacological options in pain management such as patient education, music, hypnosis, relaxation training, distraction, biofeedback, humor, massage, aromatherapy, reflexology, acupuncture, therapeutic touch, and transcutaneous electrical nerve stimulation are regarded as new ways to counter the side effects of ESWL.^[9]^ Among these methods, music therapy has been widely applied due to its sedative and tranquilizing benefits.^[10,11]^ Few studies have investigated the role of music in the control of pain and anxiety after ESWL. Music therapy was previously used in conjunction with other anesthetics or sedatives to enhance their effectiveness. Yilmaz et al reported that midazolam in combination with music places patients in a more comfortable state of mind.^[6]^ However, Cepeda et al showed that music did not significantly reduce the amount of anesthetics used.^[12]^ Though prior reviews show that music appeared to decrease anxiety and pain in urologic surgery.^[13]^ The majority of the studies combined music with an anesthetic or opioid prescription which may affect the true effect of music therapy. Moreover, only few studies performed direct comparisons and the high-quality studies is lacked.

Therefore, the present evidence is insufficient for evaluating the impact of exclusive music therapy (or in combination with low-grade analgesic) on anxiety and pain control. In past 5 years, more and more high-level prospective studies on music in ESWL have emerged. Therefore, we conducted a systematic review and meta-analysis of randomized controlled trials (RCTs) to investigate the strength of the evidence for the use of music during ESWL procedures.

## 2 Materials and methods

Our systematic review and meta-analysis followed the guidelines of the Preferred Reporting Items for Systematic Reviews and Meta-analysis statement and the Cochrane Handbook for Systematic Reviews of Interventions.^[14]^ Ethical approval and patient consent were not required because all analyses were based on previously published studies.

### 2.1 Literature search and selection criteria

PubMed, Embase, Web of Science, EBSCO, and the Cochrane library from up to May 2020 were searched systematically with the following keywords: “music,” “urolithiasis,” “musical therapy,” “pain,” “anxiety,” and “shock wave lithotripsy.” The list of retrieved studies and relevant reviews were assessed manually, and the process mentioned above was performed several times to ensure that all eligible studies were included.

Inclusion criteria were as follows:

(1) RCT study design,
(2) comparison between ESWL with music (or with low-grade analgesics) versus ESWL with no intervention (or only with low-grade analgesics),
(3) adequate reporting of data provided for analysis, and
(4) full text in English.

Full electronic search strategy for PubMed: #1 “Shock wave lithotripsy” [Mesh] #2 Shock wave lithotripsy [Title/Abstract] #3 #1 OR #2 #4 “Urolithiasis” [Mesh] #5 Urolithiasis [Title/Abstract] #6 #4 OR #5 #7 “randomized controlled trial” [Mesh] #8 randomized controlled trial [Title/Abstract] #9 #7 OR #8 #10 “musical therapy” [Mesh] #11 musical therapy [Title/Abstract] #12 #10 OR #11 #13 “pain” [Mesh] #14 pain [Title/Abstract] #15 #13 OR #14 #16 “anxiety” [Mesh] #17 anxiety [Title/Abstract] #18 #16 OR #17 #25 #12 OR #15 OR #18 #26 #3 AND #6 AND #9

### 2.2 Data extraction and outcome measures

Baseline information that was extracted from the original studies and included the following: first author, published year, number of patients, patient age and gender distributions, description of calculus, and detailed characteristics of the studies. Data were independently extracted by 2 investigators. Discrepancies were resolved by consensus.

### 2.3 Quality assessment of individual studies

The methodological quality of each RCT was assessed according to the Jadad Scale, which comprises of the following three evaluation elements: randomization (0–2 points), blinding (0–2 points), and dropouts and withdrawals (0–1 points).^[15]^ One point was awarded for each element that was conducted and appropriately described in the original article. The total score varied from 0 to 5 points. An article with a Jadad score of ≤2 was considered to be of low quality while a Jadad score of ≥3 indicated a high-quality study.^[16]^

### 2.4 Statistical analysis

Risk ratios (RR) with 95% confidence intervals (CIs) were calculated for dichotomous outcomes. Heterogeneity was evaluated using the I^2^ statistic, with I^2^ > 50% taken to indicate significant heterogeneity.^[17]^ Sensitivity analysis was performed for evaluating the influence of a single study on the overall estimate by omitting one study in turn or performing subgroup analysis. The random-effects model was used for meta-analysis. Owing to the limited number of included studies (<10), publication bias was not assessed. Statistical significance was accepted at *P* < .05. All statistical analyses were performed using Review Manager Software Version 5.3 (The Cochrane Collaboration, UK).

## 3 Results

### 3.1 Literature search, study characteristics, and quality assessment

**Figure 1:**
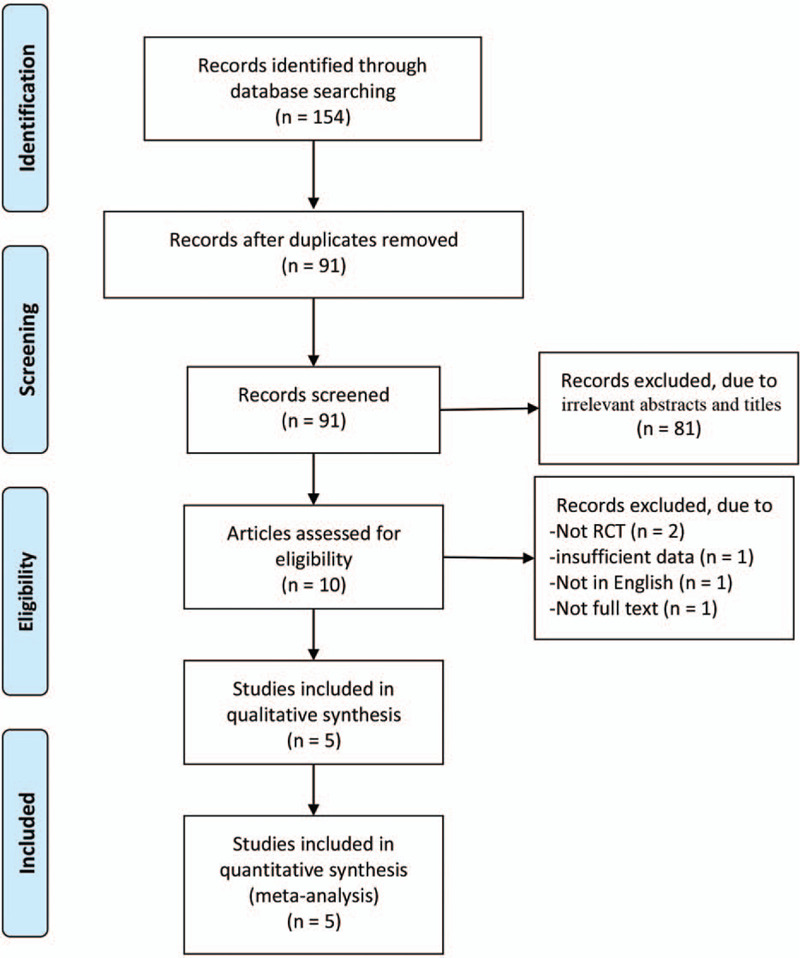
Flow diagram of study searching and selection process.

In total, 154 articles were initially identified from the databases. After removing duplicates, 91 articles were retained. Then 81 studies were excluded from our study due to unrelated abstracts and titles. We also excluded from our analysis: 2 articles for their study design (not RCT), 2 articles for insufficient data, 1 article for publication not in English, and 1 article for unavailable full text. Finally, 5 RCTs that satisfied the inclusion criteria were enrolled in this meta-analysis.^[1,18–20]^ The article selection process followed the Preferred Reporting Items for Systematic Reviews and Meta-analysis statement (Fig. 1). The baseline characteristics of the 5 included studies are shown in Table 1. The studies in our meta-analysis were published between 2016 and 2020 and the total sample size was 580. Karalar et al^[21]^ administered diclofenac 75 mg before the ESWL procedure while Khoury et al used one kind of nonsteroidal anti-inflammatory as their standard pretreatment(which nonsteroidal anti-inflammatory did not indicate).^[20]^ Patients were able to choose the type of the music in study of Cakmak et al^[18]^ and Gezginci et al^[1]^ and Karalar et al^[21]^ used classical music as the intervention. Khoury et al allowed the patient to choose the content they listened to and if nothing was chosen then random music was played. Finally, Çift et al^[19]^ divided their study population into 3 groups with 1 group listening to art music, another group listening to classical music, and the final group was allowed to choose the type of music they liked.

**Table 1:** Characteristics of included studies.

			Music group					Control group					
No.	Author	Year	Number (n)	Age (Mean)	Male (n)	Calculus location (Renal /Ureteral)	Method	Number (n)	Age (Mean ± SD)	Male (n)	Calculus location (Renal /Ureteral)	Method	Jadad score
1	Karalar	2016	57	47.75 ± 13.62	35	-	Turkish classical music with diclofenac 75mg	32	48.2–15.5	19	-	Diclofenac 75mg	4
2	Cakmak	2016	95	42.5 ± 12.7	66	56/39	Music selected by patients	105	43.3 ± 12.8	71	62/43	None	4
3	Gezginci	2017	40	-	35	21/19	Music selected by patients	40	-	37	19./21	None	5
4	Khoury	2020	30	51.5 ± 17.4	21	25/5	Music selected by patients	31	52.6 ± 16.1	22	25/6	One NSAID	3
5	Çift	2020	90	36.8,10.48	33	90/0	(Turkish art: Western classical: selected) 30:30:30	60	38.2 ± 12.93	68	60/0	None	3

Jadad scores of the 5 included studies ranged from 3 to 5 and all 5 studies were high-quality RCTs based on the quality assessment. None of the included studies reported side-effect.

### 3.2 Pain control

All included RCTs reported the pain level using a visual analog scale at the end of the session (0 = no pain, 10 = maximal possible pain).^[22]^

**Figure 2:**
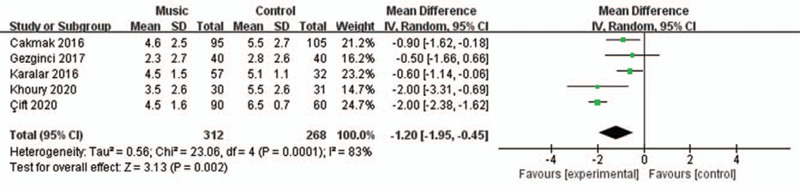
Forest plot for the meta-analysis of pain control.

We applied a random-effects model for the analysis of this outcome. The results indicated that compared to the control group, listening to music significantly reduced pain (RR = –1.20, 95% CI = –1.95 to –0.45, *P* = .002) with a heterogeneity of I^2^ = 83% (*P* = .0001, Fig. 2).

### 3.3 Anxiety reduction

Data of 4 RCTs was adequate for the analysis of anxiety reduction,^[1,18,19,21]^ in which a self-reported anxiety scale was used (State-Trait Anxiety Inventory, STAI) where a higher score indicated higher anxiety levels.

**Figure 3:**
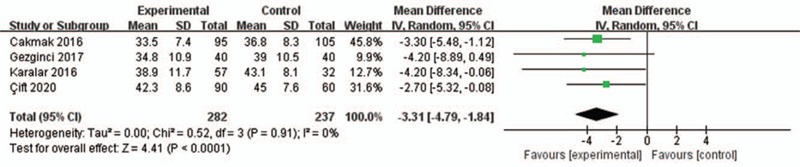
Forest plot for the meta-analysis of anxiety reduction.

The random-effects model was also used for the analysis of this outcome. The results indicated that compared to the control group, listening to the music significantly reduced the level of anxiety in patients (RR = –3.31, 95% CI = –4.97 to –1.84, *P* < .0001) with an insignificant heterogeneity among the studies (*I*^2^ = 0%, *P* = .091, Fig. 3).

### 3.4 Satisfaction, willingness to repeat, and stone clearance rate

Compared with control intervention, music therapy gave patients more satisfaction with the treatment and increased willingness to repeat according to the studies of Cakmak et al^[18]^ and Çift et al also demonstrated there was no significant difference in the stone clearance rate.^[19]^

### 3.5 Sensitivity analysis

**Figure 4:**
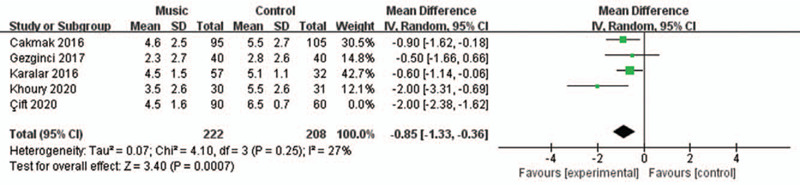
Forest plot for the meta-analysis of pain control after the sensitive analysis.

A sensitivity analysis was performed to evaluate the stability of the results due to a significant heterogeneity in the outcome of pain control among studies. After removing one study at a time, we found that the heterogeneity was mainly a result of data from Çift et al.^[19]^ However, after removing this study, the results from our analysis remained similar (RR = –0.85, 95% CI = –1.33 to –0.36, *P* = .0007) with an absence of heterogeneity (*I*^2^ = 27%, *P* = .25, Fig. 4).

## 4 Discussion

ESWL is currently the most accepted and frequently used method for the treatment of kidney stones since in clinical practice.^[23]^ In addition to the state of the kidney and stone characteristics, patient compliance also determined the success of the ESWL. Thus, many adjuvant therapies were investigated to reduce the pain and anxiety levels throughout the procedure to improve compliance. Demir et al showed that using medication to decrease the discomfort is important for repeated sessions and success of ESWL.^[24]^ This led to the widespread use of anxiolytic and analgesics to control anxiety and pain in patients. Nevertheless, these pharmacotherapies are not only costly but also lead to side-effects like respiratory depression, hypotension, and allergic reactions. As a result, nonpharmacological methods became hot topics in recent years. Listening to music has been accepted as the least invasive alternative treatment for many diseases.^[25]^ Current studies have shown that music played during ESWL can significantly help reduce the analgesia like Alfentanil needs.^[26]^ However, the effect of using music alone to reduce the patient's discomfort remains unclear. Herein, we conducted a meta-analysis to investigate the impact of music therapy during ESWL on anxiety and pain control of patients.

Our results suggested that listening to music could significantly reduce pain and anxiety. Firstly, music itself can reduce the related discomforts by modifying the neurophysiological response.^[27]^ Current theories suggest that music therapy modulates pain perception by reducing the delta-band activity in the cingulate gyrus and increasing the gamma-band activity in somatosensory brain structures at different processing stages of pain.^[28]^ Secondly, music can mask the disturbing noise and vibrations from the ESWL device, which projects negative feelings on the patients.^[27]^ Allowing patients to reach this comfortable state improves the positioning of patients and the targeting of the device and delivery of energy. Although there is no significant stone clearance rate in one single session in study of Çift et al, patient satisfaction was improved and patients were willing to repeat the sessions.^[19]^ Similar results were demonstrated by Cakmak et al.^[18]^

The overall heterogeneity in pain control was initially attributed to the clinical heterogeneity from the study by Çift et al.^[19]^ However, the reduction in pain experienced by patients remained significant after the exclusion of this study. The heterogeneity is mainly from its study design as patients were divided into 3 groups in their study, in which they received Turkish art music, Western classical music, or the patient's music of preference. In contrast, other studies exposed all participants to the same music genre. The data indicated that patients who listened to the music of their preference had a better effect.^[19]^ Another method to increase the therapeutic effects of music was demonstrated by Karalar et al.^[21]^ Music therapy with noise-canceling headphones was more potent for pain and anxiety reduction since it effectively decreased environmental noise from the ESWL device. Furthermore, other nonpharmacological distraction methods were also investigated in the studies of Gezginci et al.^[1]^ and Khoury et al.^[20]^ The traditional stress ball and modern visual media also seemed to have a positive effect on reducing procedure-related anxiety and pain. Nevertheless, music still outperformed these alternative methods.

To the best of our knowledge, this is the first systematic review and meta-analysis investigating the impact of music on the patient's level of anxiety and pain control during ESWL. However, some limitations remained in this study. Firstly, the sample size in each study was relatively small and the types of music is limited. Secondly, different stone size, position, composition, and severity of obstruction may affect the findings and is worth investigating in future studies. Thirdly, the final outcomes seemed to be biased by different types of music and patient preferences. Lastly, missing and unpublished data also led to bias in the true impact of music.

## 5 Conclusion

In conclusion, the results of this systematic review showed that listening to music could reduce the patient's pain and anxiety significantly with increased satisfaction and willingness to repeat the procedure.

## Acknowledgments

Authors thank Ling Chen, PhD, for her help on the selection of the thesis topic.

## Author contributions

**Conceptualization:** Wu-Ran Wei, Zhenghao Wang.

**Data curation:** Zhenghao Wang, Dechao Feng.

**Formal analysis:** Zhenghao Wang, Dechao Feng.

**Funding acquisition:** Zhenghao Wang, Dechao Feng.

**Investigation:** Zhenghao Wang, Dechao Feng.

**Methodology:** Zhenghao Wang.

**Project administration:** Zhenghao Wang.

**Resources:** Zhenghao Wang.

**Software:** Zhenghao Wang, Dechao Feng.

**Supervision:** Wu-Ran Wei, Jia Wang.

**Validation:** Zhenghao Wang.

**Visualization:** Zhenghao Wang.

**Writing – original draft:** Zhenghao Wang.

**Writing – review & editing:** Zhenghao Wang.

## Abbreviations

Abbreviations: CIs = confidence intervals, ESWL = extracorporeal shock wave lithotripsy, RCTs = randomised controlled trials, RR = risk ratios.
